# Public engagement and argumentation in science

**DOI:** 10.1007/s13194-022-00480-y

**Published:** 2022-08-09

**Authors:** Silvia Ivani, Catarina Dutilh Novaes

**Affiliations:** 1 University College Dublin, Dublin, Ireland; 2 VU Amsterdam, Amsterdam, Netherlands; 3 Arché, University of St. Andrews, St. Andrews, Scotland, UK

**Keywords:** Public engagement, Responsible research and innovation, Vaccine hesitancy, Argumentation, Trust in science

## Abstract

Public engagement is one of the fundamental pillars of the European programme for research and innovation *Horizon 2020*. The programme encourages engagement that not only fosters science education and dissemination, but also promotes two-way dialogues between scientists and the public at various stages of research. Establishing such dialogues between different groups of societal actors is seen as crucial in order to attain epistemic as well as social desiderata at the intersection between science and society. However, whether these dialogues can actually help attaining these desiderata is far from obvious. This paper discusses some of the costs, risks, and benefits of dialogical public engagement practices, and proposes a strategy to analyse these argumentative practices based on a three-tiered model of epistemic exchange. As a case study, we discuss the phenomenon of vaccine hesitancy, arguably a result of suboptimal public engagement, and show how the proposed model can shed new light on the problem.

## Introduction

The European programme for research and innovation Horizon 2020 adopts the Responsible Research and Innovation approach (RRI), which “requires all societal actors (researchers, citizens, policy makers, business, third sector organisations etc.) to work together during the whole research and innovation process” (Figueiredo Nascimento et al., 2016, 12; see also Owen et al., 2012; Von Schomberg, 2013; European Commission, 2014). The idea underlying RRI is that science should be done *with* and *for* society: research and innovation should be the product of joint efforts of scientists and citizens, and should serve societal interests (Figueiredo Nascimento et al., 2016; Owen et al., 2012).

Public engagement (PE) practices in science are one of the fundamental commitments of RRI. The PE practices encouraged by Horizon 2020 aim at creating channels that facilitate communication and mutual understanding between science and society, and which promote science education and dissemination.^1^ In particular, Horizon 2020 encourages the use of *dialogical* practices, i.e., practices establishing two-way communication between scientific experts and non-experts at various stages of the scientific process (e.g., design of scientific projects and plan of research priorities; Geoghegan-Quinn, 2014).

Collaboration and responsiveness to society’s inputs in science are understood as crucial to attain *epistemic* desiderata (e.g., acquiring new knowledge) as well as *social* desiderata (e.g., better research and innovation governance, improving citizens’ living conditions). The thought is that issues at the intersection between science, policy and society need to be addressed by promoting dialogues between “co-existing worldviews and knowledge production spaces in science, society and policy” (Figueiredo Nascimento et al., 2016, 7). Diversity is seen as crucial to achieve epistemic progress: adopting a synergistic approach that allows different actors to contribute with their diverse bodies of knowledge and experiences would make it possible to raise and address new significant research questions, gather relevant data, and finally attain new knowledge. Moreover, involving different societal actors is seen as fundamental to achieve a better understanding of the challenges faced by societies, and to develop research that is sensitive to these challenges and thus able to serve societal needs.

Researchers working on science communication and science policy (including some philosophers of science) have offered insightful discussions of PE and RRI (see a 2014 special issue on public engagement in science in the journal *Public Understanding of Science*, in particular its introduction (Stilgoe et al., 2014), and a special issue on RRI in the journal *Synthese*, in particular its introduction (Carrier & Irzik, 2019)). They are well aware that, while there may be excellent reasons to engage in PE, there are also costs, obstacles, and risks involved.

This paper offers a contribution to these ongoing debates. We inquire whether, how, and to what extent these dialogical practices can promote the attainment of epistemic and social desiderata. A number of studies have defended RRI and PE by highlighting their beneficial impact on scientific research and technological innovation (e.g., by fostering the emergence of new research topics and decreasing scientific misconduct, Mejlgaard et al., 2018, 3; see also Mejlgaard et al., 2016). But the desirability of the approach has also been questioned; it seems that PE is often not fully delivering on its promises (Scheufele, 2011). There is also the concern that public engagement has become primarily a buzzword, and that the associated rhetoric is running ahead of actual practices (Weingart et al., 2021). Moreover, engaging in PE practices is often perceived as impairing freedom in research (Carrier & Gartzlaff, 2020, 157; see also Small & Mallon, 2007). The alignment of research and technological innovation to societal values, needs, and expectations is seen as an external control hindering creativity and as an obstacle to the flourishment of research and innovation.

In view of these tensions, we ask: is PE indeed desirable and possible, and if so, how? What are the risks, costs and pitfalls associated with PE, and how might they be minimized? Given Horizon 2020’s emphasis on the use of dialogical practices, our analysis is based on the idea that PE can be understood as corresponding to argumentative practices occurring in networks in which societal and scientific actors can offer, receive, and exchange different types of (epistemic and non-epistemic) resources. If PE is thus conceived, it can be analysed using a three-tiered model of epistemic exchange, presented in (Dutilh Novaes, 2020), which distinguishes between: (1) networks of potential exchanges, (2) the choices that actors make among potential exchange partners, and (3) the exchanges properly speaking. This model allows for a systematic investigation of PE practices in their different facets, and offers tentative answers on how PE practices can be improved.

The paper is structured as follows. In Sect. 2, we present the benefits but also the costs and risks of promoting dialogical practices between scientific and societal actors. Section 3 introduces the three-tiered model of argumentative engagement. In Sect. 4, we extend this model to PE. In Sect. 5, we apply this model to a case study, namely the phenomenon of vaccine hesitancy, relying substantially on the work of M. Goldenberg (Goldenberg, 2021), but approaching her findings from the perspective of the three-tiered model.

## Upsides and downsides of PE

PE includes a myriad of practices, which may differ in their organizing entity (national or local governmental body, museum, industry, academia etc.), settings (local or transnational cooperation), target groups (lay public, stakeholders, experts), main purpose (education, awareness raising, knowledge co-production), and time span (from single events to projects running over several years) (PE2020, 2015, 8–9). Moreover, while some practices involve one-way communication, in which information flows from one group to another, others involve two-way communication, in which dialogue is encouraged and information flows in both directions. The *Public Engagement 2020* project (PE2020) provides an analysis of various PE activities that have been implemented between 1992 and 2018 and, based on the direction of the information flow and purpose of the activities, suggests classifying them into 5 main categories (Rask et al., 2018, 20–21):

1) Public communication: one-way communication from sponsors of initiatives to public representatives, such as public hearings and awareness raising initiatives. It aims at informing and educating citizens, and typically does not include mechanisms to handle and integrate feedback from the public. An example is the *Nanodialogue Project* (Rask et al., 2018, 42): one of its main goals was to develop channels to communicate research developments in nanotechnologies and nanosciences to the general public.

2) Public consultation: one-way communication from citizens to sponsors, such as citizens’ panels and focus groups. Public consultations are initiated by sponsors that seek to gauge the public’s opinions on a certain topic so as to inform decision-makers, but no two-way dialogue is involved. For instance, the *VOICES* Project was a year-long (2013–2014) public consultation initiative which collected European citizens’ preferences and expectations regarding European research priorities (Rask et al., 2018, 42).

3) Public deliberation: two-way communication, such as citizen juries and consensus conferences, that aims at group deliberation on policy issues. It involves dialogues between the sponsors, scientists, and the public in which information is exchanged, primarily in the form of arguments. The *World Wide Views on Global Warming* is an example of global citizen deliberation, which focused on producing policy recommendations regarding climate change (Rask et al., 2018, 100).

4) Public participation: two-way communication, such as co-governance and direct democracy mechanisms. It aims at providing citizens with decision-making power on policy issues. It involves dialogues between the sponsors and the public in which information is exchanged. An example of public participation is the *We the Citizens* project, a PE initiative that enabled citizens to contribute to the design of the Irish Constitutional Convention (Rask et al., 2018, 71–72).

5) Public activism: one-way communication from citizens to sponsors, such as demonstrations and protests. It aims at influencing decisions by creating awareness. Unlike public consultation, the communication is not initiated by the sponsors, but by the public (Rask et al., 2018, 21–22; see also PE2020, 2015). For instance, the *Let’s do it!* campaign was organized by a global non-governmental organization and aimed at raising awareness on sustainability and organizing activities to remove illegally dumped waste from the environment.

Horizon 2020 encourages both one-way and two-way communication practices, but emphasizes PE that applies two-way communication methods.^2^ PE2020’s analysis of recent trends reveals that “there has been a shift of PE from traditional models of public communication and consultation, in which dialogue between decision makers and the public is narrow and restricted, to public deliberation in which such dialogues are intensive and influential” (Rask et al., 2018, 112). Indeed, nearly half of the PE projects analysed by PE2020 were public deliberation initiatives (Rask et al., 2018, 51; see also Engage2020, 2015). Moreover, the majority of the cases studied—even the ones that had public consultation or public communication as main objectives—involved two-way or multiple-way communications (Rask et al., 2018, 52).

The recent shift towards a more responsive and collaborative PE approach was motivated by, among other factors, the idea that two-way communication PE is the most efficient strategy to ensure that research and innovation will successfully handle societal challenges (Geoghegan-Quinn, 2014). However, some studies have detected a number of barriers that may lower PE’s effectiveness. For instance, PE2020 identifies some managerial obstacles (e.g., difficulties in managing conflict between participants and promoting fruitful communications), as well as problems related to impact, i.e., low absorption of PE outcomes by decision makers (Rask et al., 2018, 108–109; see also Rask, 2013). Against this background, we now present a systematic overview of the benefits, risks, and costs of PE practices in science. We start with the good news about PE, i.e., why it seems like a very good idea.

Decisions taken in scientific contexts can have a deep, disruptive impact on society (for better or worse). Prioritizing certain research questions over others can determine in which direction society will develop; the design we opt for when working on a new technology can have consequences for how comfortable citizens’ living conditions will be. For instance, choices on how to develop a vaccine for a certain disease may have an impact on whether target groups will actually have access to and benefit from the technology.^3^ PE practices are then to be recommended if it is thought that decisions in science should not be taken only “by a minority elite” (Douglas, 2005, 156), but should instead be the result of processes in which different stakeholders have the opportunity to participate (following the basic tenets of deliberative democracy). In the European Union, Horizon 2020 encourages PE practices that aim at empowering EU citizens and allowing them to have a say on whether and how research and innovation will disrupt their lives (in particular in the case of policy-relevant science such as public health or climate research). A further and related reason for bringing citizens to the forefront of scientific decisions is that, since they provide funds for research and innovation as taxpayers, they presumably have the right to have a say on how these funds are spent (Feyerabend, 1978; Figueiredo Nascimento et al., 2016).

Furthermore, fostering inclusion and responsiveness in science is important if this benefits not only the members of society but also science as an institution. Integrating diverse perspectives would make it possible to detect and challenge problematic mechanisms in science, e.g., unfair funding mechanisms or sexist or racist practices that marginalize or ignore the needs of certain groups. A fitting example is the genSET project, which brought together European science leaders, gender experts, and stakeholder institutions to discuss the gender dimension in science and produce action plans to increase women’s participation in science.^4^ PE practices can then set off an ameliorative process whereby the dynamics and power structures of science are revised and refined (Stilgoe et al., 2014, 6; see also Wynne, 2006). In such cases, participants to PE can then provide value-related resources (e.g., ethical values, ideals, ambitions, needs) that can enrich and improve scientific practices, and facilitate the attainment of social desiderata.

Moreover, the inclusion of different societal actors in some phases of research may be epistemically beneficial to science, including the active participation of citizens in producing scientific results (in what is known as *citizen science*; Gura, 2013). The different bodies of knowledge that specific groups may have because of their social locations and experiences can qualify as valuable epistemic resources that can enrich the discussion and lead to better science. Having access to these bodies of knowledge may be crucial to tackle certain scientific challenges (European Commission, 2009; Geoghegan-Quinn, 2014). Several studies have shown the importance of integrating so-called *local knowledge*, understood as information over a specific situation/condition given one’s own personal involvement or experience of that situation (e.g., Barrotta & Montuschi 2018; Douglas, 2005). Given their experiential access to certain situations, citizens may be precious sources of information, and engaging with these resources may be essential to make epistemic progress and avoid errors (Barrotta & Montuschi, 2018). For instance, involving long-COVID patients is seen as crucial to gather relevant information on the condition, given their unique experiential knowledge (Ladds et al., 2020).

PE is often also seen as a promising strategy to regain or increase public trust in science and scientific institutions. Recent studies have shown that, although public trust in science remained consistently strong over the last decades (Funk et al., 2019), some segments of the population have become more distrustful of science (e.g., conservatives in US; Gauchat, 2012). (Some authors have even gone as far as proclaiming the ‘death of expertise’; Nichols, 2017.) Moreover, trust may vary across domains of research (e.g., climate change and vaccination trigger more skeptical views than other domains), and depending on how research is funded (e.g., there is more support for publicly funded vs. privately funded stem cell research; Critchley, 2008; see also Maxim et al., 2013, 691). Various factors have been proposed to account for this distrust, such as unjust treatment of minority groups in scientific practice (e.g., women’s exclusion from clinical trials), scientific misconduct, and increasing commercialization of science (de Melo-Martín & Intemann, 2018, 96–113; Goldenberg, 2021). This research also shows that having open public access to data may increase trust in scientific research (Funk et al., 2019). RRI’s approach to PE is a response to public distrust in science (Carrier & Irzik, 2019): PE is expected to bring science closer to society by making research and innovation more accessible, to promote transparency and accountability, and to show that scientific institutions have a genuine interest in addressing the public’s needs and ambitions.

Thus seen, PE may well be a promising strategy to overcome misunderstanding and avoid non-experts’ negative reactions towards science. By directly involving non-experts in dialogues about scientific decisions, PE may enable citizens to have access to scientific information, avoid confusion caused by overabundance of (mixed or unreliable) information that other non-official channels may create (Nguyen & Catalan, 2020), and promote conversations with scientists who are involved in the research. The thought is that being open to discussing the risks and limitations of their own research with a diverse audience may enable researchers to (re)gain the public’s trust (Berg, 2008). Moreover, research on science communication suggests that lay people’s reactions to scientific uncertainty may depend on the source. For instance, Jensen and colleagues (2017) conducted a study on how lay people react to uncertainty in cancer research, and their findings show that people expressed less fatalism about cancer prevention when those communicating uncertainty were the very researchers involved in the study, when compared to communication by scientists not directly involved.

However, there are also reasons to be concerned about PE. For starters, extensive PE may limit the time and resources that scientists can employ in their research. Studies reveal that scientists often perceive PE as an administrative burden diverting energy and resources that could be better employed elsewhere (Carrier & Gartzlaff, 2020, 163; Small & Mallon, 2007). Moreover, being involved in these practices means that scientists are personally exposed to the public, and may experience undesirable consequences such as personal threats. For example, during the COVID-19 pandemic, researchers working on SARS-CoV-2 often received political and ideological attacks (e.g., Hallal, 2021). (The experience of Marc van Ranst, Belgium’s most prominent scientific figure during the COVID-19 pandemic who had to live in a safehouse for a month in 2021 after receiving death threats, is a case in point.) A question that needs to be addressed then is whether it is desirable for scientists to be thus exposed to the public (including ill-humored citizens), and whether strategies to protect them from possible dangers should be developed.

A further reason to be concerned about PE is that communication of scientific results—in particular in cases of uncertainty—may have some undesirable effects. Some studies have shown that the communication of scientific uncertainty to non-experts may elicit negative responses in citizens, such as fatalism and panic. Indeed, it seems that uncertainty may provoke reactions of fear and outrage among lay people (Johnson & Slovic, 1998); they seem to view scientific uncertainty as a signal of the incompetence of scientists (Johnson & Slovic, 1995). The psychological literature reports mixed results on the effects of communicating scientific uncertainty to the public: while some studies report a detrimental effect that communication of scientific uncertainty may have on public trust in scientists, others did not find such effect (e.g., Retzbach & Maier, 2015). If, in some circumstances, communication of scientific results may elicit negative reactions in the public, we need to understand whether (and under which circumstances) there may be a risk that PE will further contribute to the spread of these negative reactions.^5^

Going in the other direction (from public to scientists), although the inclusion of epistemic and social resources provided by citizens may in some cases be epistemically and socially valuable, it may also be undesirable in some other cases. Some pieces of information may be irrelevant or even noxious, i.e., they may be useless for research or, if integrated in scientific decisions, may seriously undermine the prospects of achieving epistemic desiderata. For instance, Carrier and Gartzlaff (2020) conducted interviews with scientists involved in PE activities, which revealed that citizens often have trivial or impossible expectations; integrating them in scientific decisions may result in fruitless inquiries. The resources provided by citizens may also lower the chances of achieving social desiderata, for example if their integration in scientific decisions leads to the perpetuation of unjust power dynamics (i.e., if the interests of more vocal, influential groups are prioritized over those of other members of society).

There are also various challenges pertaining to implementation. Several studies show that scientists face practical challenges when planning, organizing, and implementing PE practices. For instance, researchers often lack incentives to be involved in PE practices (e.g., benefits for career advancement), and they rarely receive training on how to engage with the public (Carrier & Gartzlaff, 2020). Tellingly, it seems that most researchers are still not familiar with the RRI approach (European Commission, 2014). In a recent survey, only 26% of the EU-funded researchers interviewed indicated familiarity with RRI (Bührer et al., 2017, 10; see also Mejlgaard et al., 2018, 3). These results show that the information flow from EU institutions to researchers is wanting, and that the integration of RRI into EU policies and practices is still incipient (Novitzky et al., 2020). Moreover, increasingly vague and inclusive definitions of ‘engagement’ as well as of the relevant ‘public’ stand in the way of de facto effective interventions (Weingart et al., 2021).

There are thus reasons to be enthusiastic as well as reasons to be concerned about PE. On the one hand, PE may be an effective strategy to reach a number of epistemic and social desiderata, e.g., producing scientific knowledge, regaining public trust, and conducting socially relevant research. On the other hand, it may also obstruct the attainment of these desiderata by bringing along costs and risks. What is required now is a realistic assessment of these different possible outcomes. RRI’s policies regarding PE seem to be based on what may be described as a ‘Millian’ conception of argumentation (in a reference to J.S. Mill), according to which stronger arguments eventually prevail as the truth ‘shines through’. From this perspective, promoting dialogical engagement on scientific matters among societal and scientific actors with different bodies of knowledge, levels of expertise, values, and needs, will (typically) be beneficial, i.e., it will lead to epistemic progress.^6^ But this Millian conception is arguably an overly optimistic view of argumentative exchanges; very often, stronger arguments simply do not seem to prevail over weaker ones (Dutilh Novaes, 2020).^7^ As such, it must be further developed if it is to account for the actual complexities involved in PE.

## A three-tiered model of epistemic exchange

In this section and the next, we offer a systematic analysis of the costs, obstacles, and risks associated with PE, following the three-tiered model of epistemic exchange presented in (Dutilh Novaes, 2020). This analysis should help explain why PE seems not to be consistently achieving its stated goals (Wynne, 2006; Scheufele, 2011; Stilgoe et al., 2014), and shed light on which factors may promote or hinder the achievement of these goals. What justifies the application of this model is the observation that PE may be suitably conceived as a form of *argumentative exchange* between scientists and members of the public, where knowledge and other epistemic resources (including value-related resources) are exchanged in both directions (from scientists to the public but also from the public to scientists). This is particularly true of the third category of PE described in Sect. 2, namely ‘public deliberation’, but the notion of argumentative exchange arguably captures at least part of what goes on in the other categories. After all, when scientists inform the public (first category, public communication), scientific knowledge is communicated in the form of statements supported by evidence and arguments; similar considerations apply to the other categories. At any rate, as noted above, Horizon 2020’s focus has been on encouraging PE that applies two-way communication methods; nearly half of the PE projects analysed by PE2020 were public deliberation initiatives, and most others included two-way or multiple-way communication (Rask et al., 2018).

In the early days of PE, the predominant conception was still that of an asymmetric relation where knowledge only flowed from the scientific experts towards the public (in what is known as the ‘knowledge deficit’ model of PE; Stilgoe et al., 2014; Goldenberg, 2021). Since then, conceptions of PE have moved towards dialogical models where the public is no longer seen as a passive recipient of scientifically produced knowledge, but rather as an active conversational partner; in particular, this dialogical conception of PE is one of the cornerstones of Horizon 2020, as noted above. (Whether this commitment is also sufficiently reflected in *practices* of PE, i.e., whether citizens participating in PE practices are actually treated as valuable conversational partners, is a different and important matter.) This switch is in part motivated by deliberative democratic ideals, especially the thought that citizens should *participate* in science governance. Moreover, it is now increasingly recognized that the public also has valuable epistemic resources to offer to scientists, despite the (presumed) asymmetry in scientific expertise. For these reasons, proponents of PE and RRI emphasize that science should be done not only *for* society but also *with* society (Carrier & Irzik, 2019).

As mentioned above, this dialogical conception of PE seems to be based on the Millian ideal of a ‘free exchange of ideas’: a process whereby participants have the opportunity to improve their epistemic positions through rational argumentative engagement with those who have different perspectives and views to offer.^8^

[Man] is capable of rectifying his mistakes, by discussion and experience. Not by experience alone. There must be discussion, to show how experience is to be interpreted. Wrong opinions and practices gradually yield to fact and argument; but facts and arguments, to produce any effect on the mind, must be brought before it. (Mill, 1999) (p. 41).

However, the free exchange of ideas is hindered by various factors such as structural power relations and cognitive and social tendencies (Dutilh Novaes, 2020), so much so that it is not a given that wrong opinions and practices will “gradually yield to fact and argument”. The three-tiered model of epistemic exchange presents a more realistic account of epistemic exchange through argumentation, which incorporates many of the Millian insights but goes further in terms of explaining the costs, obstacles, and risks of engaging in argumentative exchanges. It helps us better understand why unfounded opinions and practices do not always “gradually yield to fact and argument”.

This model was inspired by a framework known as *Social Exchange Theory* (SET) (Dutilh Novaes, 2020). This is a framework developed by sociologists and social psychologists that seeks to explain human social behavior in terms of processes of exchange between parties involving costs and rewards, against the background of social networks and power structures (Cook, 2013). It was originally developed in the late 1950s and early 1960s under the influence of research in economics (rational choice theory) and psychology (behaviorism), but also inspired by anthropological work by Malinowski, Mauss, and Lévi-Strauss (Cook, 2013). SET is an influential and empirically robust framework, which has been used to investigate a wide range of social phenomena (such as romantic relationships, business interactions, trust in public institutions, among many others). In particular, and relevant for our purposes, it has been extensively used to investigate interpersonal communication (Roloff, 2015). The SET models are neither purely descriptive—as they rely on certain normative assumptions such as that agents seek to maximize rewards and minimize costs—nor purely normative, given that they incorporate experimental findings as well as extensive observational data. Moreover, SET combines a first-person perspective, which explains and predicts choices that individuals make between different potential exchange partners, with a third-person perspective, which focuses on structural features of these exchange networks.

The three-tiered model of epistemic exchange adapts insights and results from SET to exchanges that are specifically *epistemic*, that is, when what is exchanged are epistemic resources such as knowledge, evidence, information etc. (see Dutilh Novaes (2020) for further details on how the three-tiered model emerges from SET). The model allows for a detailed analysis of the conditions under which successful epistemic exchange may occur or fail to occur. There seem to be two preliminary stages that determine whether a given agent will be in a position to engage in fruitful epistemic exchange: the *networks* that determine which sources and which epistemic resources an agent is exposed to at all; and the *contrastive choices* that agents must make regarding which contents and sources to engage with (among those she is exposed to). Thus seen, the three stages for epistemic exchange are:

1. **Attention/exposure**. The first stage consists in establishing whether two people are potential exchange partners at all, given the relevant opportunity structures for epistemic engagement. In simpler terms: who is in an agent’s network of potential contacts? Who is in a position to attract the attention of others? It may be that potential lines of communication are cut, say in the case of structural censorship or echo chambers (Nguyen, 2020). But it may also be that so many signals are being broadcast that many different sources are all competing for the receiver’s attention, in a so-called ‘attention economy’ (Franck, 2019).^9^
2. **Choosing whom to engage with**. The next stage involves making choices against the background of possibilities for exchange as determined by the relevant opportunity structures. Typically, there will be a number of options, and given limitations of time, attention etc., contrastive choices will have to be made. Among those who have caught my initial attention, whom do I further engage with (if at all)? It is at this point that considerations of *trustworthiness* (Hawley, 2014) come into play (among other factors). In particular, trusting someone will often entail *not* trusting someone else, especially when their respective messages conflict (Dutilh Novaes, 2020).
3. **Engagement with content**. It is only at a third stage that engagement with *content* properly speaking should occur; this is when the actual epistemic exchange in fact takes place. At this point, the receiver will reflectively (and perhaps critically) engage with the argument, evidence or content being offered, seeking to understand its substance and evaluate its cogency. In case of a positive evaluation, this may lead to a change in view for the receiver (though even at this stage the receiver may still balk at revising her beliefs). It may also lead to a mutually beneficial exchange where both arguer and addressee improve their respective epistemic stances, as posited by Mill, and in some cases even go on to create new epistemic resources together (as in Lakatos’ ‘proofs and refutations’ model of mathematical practice (Lakatos, 1976)).

**Fig. 1:**
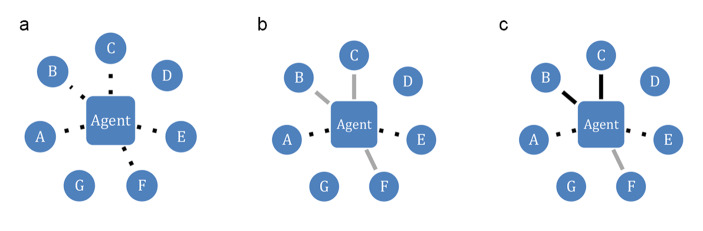
**a** Attention. **b** Trust. **c** Engagement

Figure 1 represents the three tiers. (For simplicity, here we represent a main agent and other agent around her, but the model in fact focuses on complex networks of agents who are interconnected to different degrees.)

1) **Attention**: Agent does not ‘see’ sources D and G, while other sources catch her attention (dotted lines).

2) **Trust**: Agent trusts B, C and F sufficiently (grey lines), but not A and E.

3) **Engagement**: Agent eventually engages substantively with B and C (black lines), but not with F.

Millian conceptions of argumentation tend to focus primarily on tier 3 and to downplay some of the structural obstacles to a truly free and equal ‘exchange of ideas’.^10^ Indeed, stages 1 and 2 crucially determine if and when someone will seriously engage with the epistemic resources being offered by someone else at all.^11^ Just as the original SET models, the three-tiered model is neither purely normative nor purely descriptive. It is not purely normative because it does not consider ideal or idealized agents: instead, it considers agents with limited cognitive resources, and who are highly susceptible to what Levy describes as ‘bad beliefs’ (Levy, 2022). Moreover, the model is empirically robust as it draws on decades of SET’s experimental and observational findings pertaining to exchanges more generally. However, the model is not purely descriptive or predictive either, as it seeks to *explain* the mechanisms that lead different people to engage in epistemic exchanges with some sources but not with others; this is done on the basis of a few foundational principles such as reciprocity and fairness, and by highlighting in particular the roles of attention and trust in such processes. As such, the model is perhaps best understood as an *explanatory model*, in the sense that it seeks to represent some of the causes of the target phenomenon and the mechanisms responsible for bringing it about (see e.g., Mäki, 2013). It may also lead to *prescriptive* recommendations on how to improve certain classes of epistemic exchanges (in the present case, PE exchanges, as we suggest below).

The three-tiered model may offer new insight into why PE can be so demanding and is often inefficient by means of a comprehensive account of these processes. First, a suitable relation of attention and exposure must emerge between the relevant scientists and the relevant public—which is far from obvious, given the highly saturated informational environments that we currently inhabit. Secondly, parties must make choices regarding whom to engage with, among the different possibilities: this is where considerations of trustworthiness (understood as related to both competence and benevolence) arise. Will citizens trust scientists enough? Will scientists take the epistemic authority of laypeople on certain topics seriously enough? Finally, the exchange itself requires that agents with very diverse epistemic backgrounds (scientific expertise vs. ‘lay’ expertise) find suitable means of communication. In the next section, we explore in more detail the challenges, risks and costs involved in PE following the three-tiered model.

## The three-tiered model applied to PE

We first turn to **tier 1**, pertaining to **attention and exposure**, which are relations between senders and receivers of messages emerging in networks of potential exchange partners. In environments of information saturation, a plethora of messages are being simultaneously broadcast, and it is far from obvious that the ‘right’ channels of communication will be established between knowledgeable senders and their target audiences. Indeed, different demographical groups obtain scientific information through different channels (Ipsos Mori, 2020; Wellcome Monitor, 2020, 2021). A recent example are the challenges involved in communicating scientific arguments supporting vaccines against COVID-19 to specific groups (Williams & Dienes, 2021; Burgess et al., 2021). In other words, an important challenge for PE in general is for scientific findings to be conveyed through suitable communication channels to those who might actually benefit from these findings. In effect, PE should be suitably inclusive, and inclusivity requires (among other factors) that the ‘right’ groups of people be effectively reached (see also Rask et al., 2018, 18).

These issues have arguably become more acute in recent years, with further fragmentation of the media landscape and the proliferation of ‘alternative’ information sources. Content consumers may get lost in a sea of options, where traditional, ‘certified’ sources compete for attention with new media. Moreover, current information technologies facilitate the emergence of epistemic bubbles (Nguyen, 2020), where exposure to information is restricted to certain kinds of sources and content (Jennings et al., 2021). For example, peer groups in social media platforms have been shown to provide very fertile environments for the dissemination of anti-vaccination discourse of dubious scientific value (Wilson & Wiysonge, 2020). It is difficult to redirect attention towards scientifically vetted information once people become ‘comfortable’ in these bubbles.

Furthermore, epistemic networks are typically characterized by uneven levels of connectivity for different agents and different regions of the network (Zollman, 2013). Some agents are highly connected in that they are able to attract the attention of many other agents and/or to obtain resources from them, while others remain at the fringes of the network. Indeed, different demographic groups have different levels of engagement with or access to scientific information. For example, studies report that those with no higher education degrees and/or members of less affluent groups tend to be less engaged with scientific information on COVID-19 and more hesitant about the safety and efficacy of vaccines (e.g., Ipsos Mori, 2020; Robertson et al., 2021). Moreover, levels of connectivity are typically related to prestige, which in turn also correlates with geographical location (e.g., science produced in low and middle-income countries tends to receive less attention and exposure; see Koch, 2020).

The bottom-line is that attention is a scarce and highly sought-after ‘commodity’ (Wu, 2016): it is often not clear whether lay citizens have the time, the energy or the interest to dedicate some of their limited attention resources to scientific findings. Contrary to what might be expected (at least from the point of view of the Enlightenment-inspired ideals motivating PE), citizens are not always particularly eager to engage with scientific findings, given limitations of time and attention and the competition from widely available and more ‘amenable’ content.^12^ Conversely, insofar as the attention of *scientists* must also be suitably directed towards members of the public with whom they want to engage, similar challenges arise if the latter are not accessible to the former, given the structure of the networks in question. Furthermore, attention dynamics can be tempered with—for example, by lobbying efforts from powerful stakeholders and propagandists (O’Connor & Weatherall, 2019). On occasion, what presents itself as ‘public engagement’ may well be an orchestrated campaign to defend the interests of specific individuals and groups (Stilgoe et al., 2014; Wynne, 2006).

Next, actors typically have to make **choices** among different potential exchange partners (as determined by their positions in informational networks); these choices belong to **tier 2** of the model. An agent will usually prefer to exchange with partners who have (what she perceives to be) valuable resources to share, and who are (presumably) not acting in bad faith, e.g., deliberately misleading or taking advantage of others (Cook, 2013). In other words, one of the main factors determining these choices is the (perceived) *trustworthiness* of the exchange partner, which can be understood as comprising two independent dimensions: *competence*, i.e., the potential partner is actually knowledgeable on the topic in question and is thus offering reliable information; and *benevolence*, i.e., the potential partner is not trying to deliberately mislead for their own benefit (Dutilh Novaes, 2020).

For PE to be successful, citizens must not only be exposed to scientific content through the right channels (tier 1); they must also have sufficient trust that the epistemic resources being offered are reliable and can be useful to them, and that scientists are not merely attempting to mislead the public to promote their own interests. Indeed, one of the rationales for PE in the context of Horizon 2020 is to regain citizens’ trust in scientific expertise, against a perceived erosion of said trust over the last decades (Figueiredo Nascimento et al., 2016, 28). The thought is that it is important to show that scientific institutions have a genuine interest in the challenges that society at large faces, rather than focusing exclusively on abstract topics unlikely to lead to applications that may benefit citizens (Carrier, 2017). In other words, the goal is to show that there is sufficient *alignment of interests* between the scientific community and society.

Given these observations, there seem to be three main kinds of factors that would undermine the public’s trust in science: (a) The public comes to doubt the competence of scientists. (b) The public comes to believe that scientists do not focus enough on questions that have actual implications for the public’s own immediate interests (as suggested by Horizon 2020). (c) The public comes to believe that a significant portion of the scientific community is motivated by spurious interests pertaining to e.g., monetary incentives—e.g., the ‘Big Pharma’ narrative.

Which of these kinds of reasons best explains the erosion of trust in science in recent years? While it is surely a multi-causal phenomenon, recent research seems to suggest that some of the main obstacles in fact pertain to c), that is, to the perception that scientists (as well as policy makers and the biomedical industry) are promoting their own interests at the expense of the public (Goldenberg, 2021).^13^ A 2019 editorial in *The Lancet Infectious Diseases* entitled ‘Trust Issues’ (The Lancet Infectious Diseases, 2019) addresses precisely this issue:

In the USA, the country is plagued by prescription opioid misuse fuelled by aggressive pharmaceutical marketing, the people of Flint, MI, have been without safe drinking water for 3 years, and the most basic drugs are often unaffordable because of profit-driven health care. Little wonder that some individuals question the authorities’ desire to prioritise their wellbeing. It is impossible to build trust while at the same time abusing it.

Now, if distrust in science is mainly motivated by these kinds of suspicion (which may be justified or not, insofar as these may be faults of science as such or of commercial and governmental institutions, which in turn may or may not work with the complicity of some scientific institutions and experts),^14^ it is far from obvious that PE as conceived in Horizon 2020 will be sufficient to *repair* trust. Typically, a modicum of trust must already be in place for successful PE to occur at all (even if successful iterations of PE may well have the effect of *increasing* existing trust). Indeed, it is very difficult to convince the already suspicious layperson that scientists are not in the business of taking advantage of the public (Duijf, 2021). To be clear, large portions of the population still place a fairly high degree of trust in science (Funk et al., 2019). But there are increasing vocal pockets of distrust, e.g., anti-vaccination movements, with potentially disruptive consequences for society at large. At the same time, given science’s less-than-stellar track record on some (many?) issues—medical experimentation without consent on minorities, problematic exclusion from clinical trials of certain groups—it is no surprise that certain communities remain deeply suspicious of scientific interventions (Washington, 2006; Hodge, 2012; Liu & Dipietro Mager, 2016; Goldenberg, 2021).^15^

Furthermore, if PE is truly supposed to be a dialogue between scientists and the public, then not only is it necessary for the public to attribute sufficient trust to scientists; trust must also be established in the other direction, which turns out to be a formidable challenge. Imbued with their aura of expertise, scientists often display a fair amount of arrogance towards laypeople when it comes to scientific matters (Barrotta & Montuschi, 2018; Wynne, 2006). For example, the burgeoning literature on epistemic injustice in healthcare settings shows that medical professionals often discount the testimonies of patients about their own conditions, simply because patients are not seen as sufficiently ‘knowledgeable’ (Carel & Kidd, 2014). In such cases, the kind of experiential knowledge accrued from actually living with a certain condition is not given much weight, as it lacks ‘scientific basis’. These phenomena suggest that scientists themselves often fail to attribute to the public the kind of trust and respect required for a genuine exchange of epistemic resources to occur. Indeed, the kind of responsiveness that scientists should display when engaging with the public according to proponents of PE (Stilgoe et al., 2013) is often found to be lacking.^16^

In sum, the three-tiered model of epistemic exchange posits that trust and trustworthiness are a key (though not the only) element guiding an agent’s choices (among potential partners) of whom to exchange with. Applied to PE, this observation implies that a condition for successful PE is the presence of sufficient *mutual* trust and respect between scientists and the public. In the absence of such trust, the success of PE is jeopardized, and it may well become a futile exercise (or even worse, it may lead to further trust erosion). Thus, it is far from obvious that PE can be used to *repair* trust between the scientific community and the public, given that trust itself is a crucial component for successful PE.^17^

Finally, assuming that the potential PE participants have established relations of mutual attention and sufficient trust, they can finally engage in the **exchange of content** properly speaking (**tier 3**). Scientists will convey some of their important discoveries to the relevant public, which may for example inform individual choices (e.g., whether to adopt social distancing measures or mask-wearing during a pandemic). Citizens will communicate their concerns and priorities to scientists, noting in particular aspects of their lives that may be improved by scientific research (e.g., specific medical conditions). Citizens may also provide valuable local knowledge that scientists can incorporate into their research, or be involved in citizen science initiatives. In successful instances of PE, one may speak of the co-production of knowledge through dialogue.

But what assurance do we have that these dialogues will indeed be as fruitful and productive as in this ideal scenario? Here again there are a number of obstacles that proponents of PE must contend with. The first is simply the issue of conveying complex, technical scientific findings and concepts to a lay audience who will typically not possess much scientific training. Is it possible to achieve this without excessive distortion? ‘Popularizing science’ is an issue that already preoccupied the philosopher Susan Stebbing in the 1930s. In *Philosophy and the Physicists* (1937), she examined critically the language used by scientists to explain their discoveries, especially when such explanations were presented as palatable and even entertaining to a popular audience. Stebbing worried that imprecise, impressionistic or appealing uses of language obscured the nature of scientific discoveries; given this potential for distortion, popular renditions of scientific findings could even lead to serious misconceptions. This problem is just as pressing now (if not more) as it was in the 1930s: the more complex scientific concepts become, the further removed they will be from everyday concepts, thus creating a schism that may result in insurmountable communication obstacles. Moreover, the fact that scientists often lack clear guidelines and training on PE practices (Carrier & Gartzlaff, 2020) may further exacerbate this problem (poor PE may ‘backfire’; see Paglieri & Castelfranchi, 2010).

Another related problem is how a lay person is to evaluate the soundness of scientific content offered to her, given her lack of scientific expertise. This becomes a particularly pressing problem when there is considerable dissent or disagreement among scientists on a given matter (Dellsén & Baghramian, 2020). The layperson may then resort to typical markers of excellence such as prestige, membership to respected institutions etc.—what Anderson (Anderson, 2011) describes as second-order assessment of the trustworthiness of experts—but she will typically not be in a position to evaluate the soundness of the scientific content being offered. Relatedly, she may fall prey to discourse that presents itself as scientifically grounded, but which is in fact meant to promote the interests of specific individuals or groups,^18^ such as in bogus scientific controversies motivated by spurious interests (Martini & Andreoletti, 2021). Moreover, the public may also develop unrealistic and unreasonable expectations of what scientific research can deliver, based on misunderstandings of what scientific practice is really like. Indeed, it is plausible that there will always be “a tension between serving people’s aspirations and correcting people’s expectations” (Carrier & Irzik, 2019; 6), and responsible research also involves not complying with public wishful thinking. ‘Overpromising’ may well have undesirable consequences: citizens may end up trusting scientific institutions even less as they do not deliver on what they promised (collaboration, inclusion, alignment etc.).^19^

Moreover, there are difficulties pertaining to the process of PE itself. How, concretely, does one do science *with* society? As noted above, given the presumed epistemic asymmetry between scientist and layperson, how does one ensure that the scientist suitably engages with the epistemic resources being offered by citizens? This concerns not only citizens’ epistemic resources (e.g., local knowledge and experiences), but also their value-related resources (e.g., ethical concerns, values, and needs). Scientists are not necessarily well-equipped to identify and suitably respond to the ethical, social, and economic values, concerns, and ideals of citizens. Once more, PE thus conceived should be a two-way street, so it is also incumbent on the scientist to engage in earnest with the content being offered by laypeople: the scientist should be *responsive* to what is communicated to her in PE (Stilgoe et al., 2013; Small & Mallon, 2007). This is of course much easier said than done, and even the well-meaning scientist may lack the time, energy or ability to truly engage with the resources being offered by the public.

Ultimately, the dialogues corresponding to PE are dialogues where participants enter the conversation with little common ground, with different assumptions, and with different vocabularies. Such dialogues are notoriously tricky, and the risk of miscommunication and misunderstanding is always looming large (Talisse, 2019). The challenges involved in science communication are well-known and have been extensively discussed in the literature (Fischhoff, 2013). The move to a dialogical paradigm does not really dissolve these challenges, and may in fact even intensify them: a participatory model such as PE requires a certain degree of symmetry and equality among participants (as befits the democratic values that motivate RRI), but this is in conflict with a perceived hierarchy of expertise where the scientist is viewed as occupying a higher position than the layperson. Weingart and colleagues (2021) view this tension as the inescapable paradox of the ‘engagement’ project: it remains a top-down enterprise based on an epistemic hierarchy, even as it promotes a participatory model. (This is of course a version of the much discussed but still unresolved issue of integrating scientific expertise with democratic values; Kitcher, 2011; Goldenberg, 2021.)

The analysis of the risks, challenges, and costs faced by PE practices proposed here seems to indicate that the project of PE serving as an equalizer and thus bringing science and democracy closer together may be overly optimistic. The main reason for this seems to be that PE faces a number of obstacles that undermine/weaken its potential to recalibrate existing power relations between scientists and public, and among different scientists. Tier-1 phenomena entail that different levels of attention and exposure will reflect existing social structures and thus ongoing disparities and inequalities, and tier-2 phenomena entail that the same holds for different levels of credibility and attributions of trustworthiness. As noted by Stilgoe et al. (2014, 6):

The suspicion is that such exercises do not sufficiently challenge, and so serve to reinforce, incumbent power structures. Public engagement can be seen by institutions as an opportunity not to rethink their policies and practices, but to gain trust for a predetermined approach (Wynne, 2006; Thorpe & Gregory, 2010). When institutions initiate dialogue, what might be envisaged as a potential ‘technology of humility’ (Jasanoff, 2003), capable of prompting institutions to question their governance, can become a ‘technology of elicitation’ (Lezaun & Soneryd, 2007), extracting public opinion in convenient ways.

However, this does not necessarily mean that PE practices should be completely abandoned. In the concluding section, we will argue that, much as democracy, PE is ‘the worst system except for all the others’: despite all these issues, refraining from promoting engagement between science and the public may have even worse consequences. But a substantive reconceptualization of the role of experts vis-à-vis the public and their role in policy decisions is called for. The three-tiered model offers a suitable vantage point for such a reconceptualization, as it explicitly problematizes the (perceived or real) *interests* that motivate agents to engage or not in different exchanges, against the background of existing power structures. In the next section, we look at the example of vaccine hesitancy, which reveals the many ways in which PE can go wrong.

## The model at work: vaccine hesitancy

To illustrate the explanatory power of the three-tiered model, we now turn to the phenomenon of vaccine hesitancy. Obviously, PE is crucial for public health policies, as citizens must become convinced of the pertinence and necessity of the policies and interventions proposed by governmental agencies (under the advice of scientists) in order to comply (though they may also comply solely in virtue of mandates or other coercive means). Moreover, if the values and concerns of citizens are taken into account when designing public health interventions, the likelihood of uptake also increases. This means that PE is crucial for public health research and interventions at different stages: when defining research agendas, when disseminating scientific findings, when implementing measures etc.

Vaccine hesitancy is a case of mismatch between what the scientific consensus recommends and the decisions taken by certain citizens: while the science has overwhelmingly established that vaccines are safe and efficient (at least those approved by the relevant regulatory bodies), a significant portion of the public (varying per context and region) is hesitant about or outright refuses vaccination (either for their children or for themselves). Vaccine hesitancy is not a new phenomenon, but it has been intensified since the publication of the infamous 1998 article by A. Wakefield claiming to establish a link between MMR vaccines and autism. The paper has since been retracted and its scientific basis has been extensively debunked, but surprisingly (to some, at least) this has not led to a waning of vaccine hesitancy; indeed, corrective factual information seems to have modest to no effect (Goldenberg, 2021) (Chaps. 1 and 2).

In a recent book, philosopher of science Goldenberg (2021) offers an in-depth investigation of vaccine hesitancy. She starts by discussing some widely held accounts of the phenomenon: the ‘ignorant public’, the ‘stubborn mind’, and the ‘death of expertise’. According to the first, vaccine hesitancy arises from the public’s lack of familiarity with the relevant science, thus echoing the ‘knowledge deficit’ model of PE referred to earlier on. The remedy in this case would consist in providing *more* scientific information to the public, i.e., corrective factual information. This approach focuses exclusively on what we here refer to as **tier 3** processes of epistemic exchange, thus disregarding elements pertaining to attention and to the evaluation of credibility and trustworthiness of the sources. According to the second account, what prevents certain citizens from making the science-based decision to get vaccinated are entrenched cognitive biases in the human mind, which lead them to reject information that conflicts with their core convictions and values. This too is an account that leans heavily on **tier 3** processes, but it also considers **tiers 1 and 2** phenomena insofar as it includes core cultural commitments, which in turn are related to identity and group membership (and thus to exposure/attention as well as to relations of trust between in-group members and distrust towards out-group members). Finally, according to the ‘death of expertise’ account, recent decades have witnessed a devaluation of the role of scientific expertise for policy- and decision-making: experts simply no longer enjoy unconditional respect from the public (the presupposition being that they once did). In contrast with the other two, this account focuses predominantly on **tier 2** processes pertaining to the contrastive choices between different possible sources of information available.

Goldenberg convincingly argues that all three approaches fail to suitably account for the phenomenon of vaccine hesitancy, especially in that all three place the ‘blame’ squarely within the public: the public is ignorant, the public is biased, the public is mistaken in their allocations of trustworthiness and credibility. Indeed, all three are based on one-way conceptions of PE: the scientific experts provide scientific knowledge, and the public passively receives it (or not). Vaccine hesitancy would be a result of faulty *reception* of scientific knowledge on the part of the public, not of faulty *transmission* of this knowledge on the part of scientists.

In contrast, Goldenberg argues that scientists, policy makers and health care practitioners have also contributed to the increase in vaccine hesitancy by often dismissing or even ridiculing the concerns of the hesitant (in particular of mothers, in the context of childhood vaccination) (Goldenberg, 2021; Chap. 6). Indeed, they have failed to view members of the public as genuine *exchange partners* (rather than as passive recipients of knowledge), whose concerns and opinions also deserve a place in the conversation (even if PE vaccination efforts ultimately aim at persuasion, as she argues in Chap. 2). As a result, these members of the public have disengaged from these conversations and have started to look elsewhere for guidance on vaccine decisions (Goldenberg, 2021; Chap. 6). Now, this is exactly what the dialogical approach to PE developed here predicts: if participants in these conversations feel they are not being treated fairly, the tendency is for them to eventually disengage (Dutilh Novaes, 2020). Goldenberg notes in particular that Wakefield’s still ongoing appeal partially stems from his perceived ‘respect’ for the concerns of hesitant parents (Goldenberg, 2021; 156), alongside his status as a ‘maverick’ who ‘speaks truth to power’.

The failure of vaccine-related PE efforts consisting solely in offering corrective scientific information (as documented in Chaps. 1 and 2 of Goldenberg’s book) thus confirms the need to take other factors into account when theorizing about and practicing PE. Goldenberg’s own diagnosis of the phenomenon focuses primarily on what we here describe as **tier 2** phenomena, pertaining to trust. She writes:

I argue for an alternate framework to better capture the phenomenon. This framework, a crisis of trust, recasts vaccine hesitancy as a sign of poor public trust of medical and scientific institutions rather than a war on scientific knowledge and expertise. (Goldenberg, 2021, p. 14)

If members of the public do not trust medical and scientific institutions and practitioners, providing corrective factual information on the alleged risks pertaining to vaccines (e.g., on the absence of links between the MMR vaccine and autism) is simply not going to cut it. These segments of the public are already convinced that these institutions and practitioners do not have the public’s best interests at heart, and thus view any scientific argument or evidence being offered as part of systematic campaigns to mislead them (as also argued in Dutilh Novaes, 2020). The two main causes of this ‘crisis of trust’ specifically with respect to vaccines are, according to Goldenberg, scientific/medical racism, discrimination and injustice; and the commercialization of biomedical science, a.k.a. ‘Big Pharma’ (Chaps. 5 and 6). Thus, while the public may still recognize the scientific/epistemic competence of experts, they doubt their *benevolence*: no amount of scientific knowledge or information will address what they consider to be the *moral* failures of scientific/medical institutions.

What about **tier 1** phenomena, pertaining to attention? The timing is, after all, quite telling: since the end of the 1990’s, the Internet has completely changed how we have access to information (for better or worse). While vaccine hesitancy existed prior to the Internet, it seems plausible that anti-vaccine discourse receives more exposure now thanks to features of the online world. Views that in the past might have been relegated to the fringes of informational networks can now be amplified (Wilson & Wiysonge, 2020). Tech giants such as Facebook and Google are now under significant pressure to curtail the spread of vaccine-related disinformation, as it has become clear that simply countering these messages with scientific correctives is not sufficient to neutralize their reach. Moreover, on social media it is easier to find like-minded people, and the ensuing conversations are likely to lead to even more extreme versions of one’s original views (Talisse, 2019). Another interesting tier-1 phenomenon is the increased exposure that anti-vaccine discourse has acquired thanks to the involvement of *celebrities and influencers*, who use their broad platforms to broadcast their views to a wide audience. In particular, media celebrity Jenny McCarthy was for years viewed as the face of ‘anti-vaxxer ideology’ in the US. Celebrities and influencers affect both tier-1 phenomena pertaining to attention and tier-2 phenomena pertaining to trust, as their status as celebrities confers them an aura of respectability—see (Archer et al., 2020).^20^

Goldenberg deliberately focuses specifically on tier 2; she does mention the influence of, e.g., social media in the spread of disinformation about vaccines, but chooses to focus on the trust dimension. She estimates that the role of social media has already been extensively discussed by other researchers, while the role of trust or lack thereof in vaccine hesitancy remains undertheorized (see also de Melo-Martín & Intemann, 2018).^21^ Still, following the three-tiered model, attention and exposure emerge as essential elements of PE, especially considering the ‘attention economy’ (Franck, 2019): scientifically grounded information competes for the public’s attention with a wide range of other, often conflicting sources. The three-tiered model can accommodate Goldenberg’s extremely insightful analysis of the role of trust in these debates, but it is more general in that it also addresses phenomena pertaining to attention and exposure in these processes in an integrated way. The model thus shows that both aspects, attention *and* trust, need to be considered in tandem when explaining vaccine hesitancy.

To conclude, PE efforts to mitigate vaccine hesitancy must take into account these multiple factors if they are to be successful. Firstly (**tier 1**), appropriate channels for the propagation of scientifically grounded information on vaccines must be deployed:^22^ the attention of the public must be earned, especially as competition with alternative, often more palatable content is stiff.^23^ Secondly (**tier 2**), the ‘crisis of trust’ that seems to be at the root of much of the problem must be addressed (and Goldenberg has some concrete proposals in the conclusion of her book). In particular, it has been noted that some of the most successful interventions arise from face-to-face conversations between vaccine-hesitant people and their primary health care providers—but again, only if the latter adopt a respectful attitude towards the concerns of the former (Shen & Dubey, 2019; Katzman & Katzman, 2021).

Finally, it remains important to convey scientific information in accessible ways (**tier 3**). By emphasizing tiers 1 and 2 of these exchanges, we do not wish to imply that tier 3, which includes epistemic processes of updating one’s beliefs in view of incoming information, is no longer relevant. Rather, the point is to counterbalance the nearly exclusive focus on tier-3 processes in, for example, the ‘knowledge deficit’ conception of PE, according to which the strength of arguments and evidence alone will ensure that scientific information prevails. Indeed, tier-3 processes also remain a fundamental aspect of PE practices, with the usual challenges pertaining to conveying complex scientific information to the lay public. Ultimately, we still want citizens to be aware of the main scientific results on a given topic or issue so that they can make informed choices (even if these choices will not be uniquely determined by the relevant scientific findings). Our main point is that making accurate scientific information accessible to the public, on vaccines and other issues, is a *necessary* but not *sufficient* component of successful PE practices.

## Conclusions

We started with the observation that Horizon 2020 sees PE as crucial to attain epistemic as well as social desiderata at the intersection between science and society. In particular, based on the idea that diversity, collaboration, and responsiveness are essential to achieve epistemic progress and successfully address societal challenges, Horizon 2020 encourages dialogical practices involving two-way communication methods. We then investigated whether, how, and to what extent PE dialogical practices can promote the attainment of these epistemic and social desiderata. Our analysis explored the benefits, costs, and risks of PE and showed that there are reasons to be enthusiastic about PE, but also reasons to be concerned about PE’s potential to effectively promote the attainment of these desiderata.

Based on the idea that PE practices can be understood as argumentative exchanges, we have argued that Horizon 2020’s approach to PE rests on a ‘Millian’ conception of argumentation, which however constitutes an overly optimistic view of what a ‘free exchange of ideas’ can achieve. We then introduced a different framework, namely the three-tiered model of epistemic exchange, which allowed us to unpack and investigate three main dimensions of PE: 1. the importance of attention/exposure networks in which PE exchanges can occur; 2. the role of trust in determining how actors choose whom they will obtain information on scientific matters from, and get involved (or not!) in PE with; 3. the exchange of content among members of a PE network, viewed as two-way communication. Moreover, we used the three-tiered model to investigate the phenomenon of vaccine hesitancy and the role of PE therein. This case study shows that focusing on only one of the tiers (for example, the exchange of content, along the lines of the ‘knowledge deficit model’) is a misguided approach to PE.

Given all these obstacles and difficulties, are we to conclude that PE practices are ultimately just a colossal waste of time and resources, which should thus be abandoned altogether? True, PE is complicated and time-consuming. But what is the alternative? What would be the consequences of discontinuing or in any case significantly reducing investments in PE?^24^ We maintain that not doing PE at all would entail a number of undesirable outcomes, which can be recognized as such even by those who do not endorse the Enlightenment vision of the intrinsic emancipatory value of (scientific) knowledge. Firstly, it must be noted that we live in environments of increasing technological complexity, where citizens are regularly confronted with choices that pertain to scientific and technological matters (e.g., should I get a vaccine during a pandemic?). In order to be able to make informed choices and to navigate our deeply science- and technology-infused societies, a certain degree of *scientific and technological literacy* seems to be crucial. As at least some of this literacy is arguably a result of PE practices, discontinuing these practices may render citizens poorly equipped to navigate their environments. (Science education in schools is not sufficient, given the constant changes in science and technology that affect citizens throughout their lives.)

Secondly, we endorse the idea that much valuable knowledge for scientists is to be found among non-scientists, in the form of local and experiential knowledge. A large body of literature has shown the benefits of considering and integrating information provided by individuals experiencing certain conditions (e.g., inhabiting a specific area, suffering from a certain disease) in scientific decisions (Barrotta & Montuschi, 2018; Carel & Kidd, 2014) (see also the ‘citizen science’ movement; Gura, 2013; Ottinger, 2010). As a result, scaling down or discontinuing PE initiatives would mean depriving researchers of the valuable input that non-experts may provide (in some domains at least). Finally, it is by now widely recognized that scientific practices must be considered in their societal embedding (Kitcher, 2011): science is not a separate segment of society, insulated from the rest. As such, scientific practices should also be held accountable to society at large, which in turn can only occur if there is a certain degree of transparency and oversight. Crucially, PE may play an important role in the accountability of science towards society. In sum, while doing PE well is very hard, not doing PE at all would arguably be even worse. But a reflection on the gap between the ‘rhetoric’ and actual practices of public engagement is needed in order to achieve better results (Weingart et al., 2021).

Our analysis highlights the obstacles that PE practices may face at each of the levels of the three-tiered model, which need to be carefully considered in order to design successful PE practices. It underscores the need to rethink PE practices taking into account the multiple layers involved in successful epistemic exchanges. Suitable relations of attention and exposure should be in place (tier 1), and mutual trust and respect must be gained and nurtured (tier 2). These observations suggest that PE is not a challenge to be tackled by individual scientists in a piecemeal way: concentrated, systematic efforts are required. In particular, measures should be developed so that PE is not only encouraged, but also *supported* by scientific institutions. As mentioned in Sect. 2, scientists often lack the time and energy as well as adequate training to design PE initiatives, and scientific expertise and goodwill alone will not suffice to develop efficacious PE. Extensive institutional support to scientists (in terms of logistics, organization, funding etc.) should be in place in order to facilitate PE initiatives: it is unfair and inefficient to place the burden of performing PE solely (or even primarily) on individual scientists. Moreover, it is important to appreciate that some of the distrust towards scientific institutions currently observed in a number of societies seems to be the result of deliberate, orchestrated campaigns by populist political actors in order to discredit traditional authoritative sources of knowledge (Mede & Schäfer, 2020). In this context, countering the political weaponization of anti-science discourse requires much more than well-designed PE initiatives. Thus, we should manage our expectations in terms of what PE by itself can achieve against the background of political power struggles.

As we write, the world is still in the grip of the COVID-19 pandemic, the most disruptive event in world history at least since WWII. During a pandemic, the intertwinement of scientific, political and societal matters becomes even more visible and acute, so it may well be that attitudes towards PE will be significantly affected in the longer run. If anything, the pandemic has shown once again that science and society are deeply dependent on each other, so we can only hope that suitable lessons will be learned on how to improve engagement between scientists and citizens.

## Footnotes

1 Obviously, PE practices have been used in science long before the advent of Horizon 2020. For instance, participatory approaches have been employed in technological innovation (e.g., Huldtgren, 2014; Sanders & Westerlund, 2011; Buur & Matthews, 2008). However, PE practices have attracted more attention in the last decade also thanks to their inclusion in programmes such as Horizon 2020.

2 The fundamental commitment of Horizon 2020 to two-way communication is made clear in the definition of PE provided by former European Commissioner for Research, Innovation and Science Máire Geoghegan-Quinn: “PE implies a two-way, iterative, inclusive and participatory process of multi-actor exchanges and dialogues” (Geoghegan-Quinn, 2014).

3 The case of HPV vaccines discussed in (Intemann & de Melo- Martín, 2010) is a case in point: the decision to develop vaccines that required cold chain for storage and had to be administered in three doses over a period of six months lowered their effectiveness, since this design made distributing and administering them to women in developing countries—a group with very high cervical cancer mortality—extremely difficult. More recently, similar considerations apply to the different vaccines against SARS-CoV-2.

4 The project applied the Consensus Conference method (genSET, 2014, 5). Typically, Consensus Conferences bring together experts and groups of lay people to discuss topics that have been previously discussed by experts only. The genSET Consensus Conferences involved experts in different fields who had no specific expertise on issues pertaining to gender and gender-related research projects acting as the “lay panel” alongside gender researchers in the role of experts (genSET, 2014, 26).

5 PE may well help avoid or mitigate negative reactions towards science, e.g., by conveying information about scientific practices and the role of uncertainty therein, by bringing related issues under discussion, and by facilitating a better understanding of such uncertainty. In particular, PE can shape and/or reshape how people think of and react to scientific uncertainty if they come to better understand that uncertainty is an integral part of scientific knowledge production rather than a shortcoming. But a number of factors should be carefully considered when planning and implementing PE initiatives, as they may have a role in determining how lay people will react to the conveyed information on uncertainty. Some of these are: the type of uncertainty communicated (Gustafson & Rice, 2020), the degree of transparency (Driedger et al., 2021), and the way uncertainty is presented (Kelp et al., 2022). For an overview of factors determining the effects of communicating uncertainty, see (Gustafson & Rice, 2020).

6 The Millian conception has also influenced some views developed in philosophy of science on how experts should interact with each other in order to secure the objectivity of scientific research. For instance, Longino argues that what ensures objectivity is fostering intersubjective criticism among scientists, and that epistemic diversity (i.e., having scientific communities that involve researchers with different background assumptions, beliefs, and values) is essential to achieve this epistemic goal (Longino, 1990; see also Intemann, 2011). How exactly Longino’s ‘Millian’ conception of scientific practice relates to the three-tiered model discussed here is a topic currently under investigation by one of the present authors.

7 Mill is well aware that truth does not always prevail in controversies; he substantiates this claim with historical and religious examples. However, his optimistic attitude is not fundamentally affected by these considerations; he remains convinced that, despite efforts to suppress truth, it tends to reappear and, in the long run, triumph. (Mill, 1999, p. 29) He seems to suggest that the main reason for truth not to prevail in specific circumstances is *persecution* (hence the importance of free speech). By contrast, our model considers a number of other structural factors that prevent stronger arguments from prevailing.

8 More generally, PE ideals are embedded in a broader Enlightenment perspective, according to which (scientific) knowledge leads to emancipation (Carus, 2008).

9 See Dutilh Novaes and de Ridder (2021) for a discussion of scarcity vs. overabundance of information in epistemic environments.

10 Naturally, Mill’s own emphasis on freedom of speech is aimed at creating a maximally inclusive informational environment, and thus at increased exposure to various views (phenomena belonging to Tier 1). Mill mentions some factors as having a central role in making exchanges more likely to succeed, such as the importance of education and training in what he calls “negative logic” (Mill, 1999) (Chap. 2). However, he does not provide a detailed analysis of the conditions under which successful epistemic exchange may occur or fail to occur; in particular, they may fail even in contexts where (religious or otherwise) persecution is not present.

11 The model can also be understood in terms of set containment: at a given point in time, the set of people I actually engage in epistemic exchanges with is a subset of those who I deem worth exchanging with, which in turn is a subset of those who, due to our respective positions in the network, are potential exchange partners for me.

12 For instance, the Public Attitude to Science 2019 study (PAS2019) shows that although UK citizens think the public should have a role in scientific decision-making, they also express little desire for being personally involved (PAS2019, 2020, 10). However, citizens’ interest may vary depending on the topic discussed or historical contingencies. For instance, the Ipsos Mori report on the effect of the COVID-19 pandemic on public attitudes to science reveals a particularly strong appetite for scientific information regarding the pandemic, and that this appetite grew over time (Ipsos Mori, 2020, 1).

13 Recently, doubts pertaining to the scientific competence of scientists have also emerged among the public in connection with the COVID-19 vaccines, which turned out to be much less effective at preventing infection than initially hoped. While the rapid development of such vaccines, which still offer high protection against serious illness, is nothing short of a miracle, claims by public officials that vaccines would ‘end the pandemic’ have given rise to unreasonable expectations among the public.

14 We owe this point to an anonymous referee.

15 Compare the Foucauldian notion of *biopower*, which refers to the many ways in which governments exercise power over the lives and bodies of citizens (Foucault et al., 2009).

16 There are some promising recent developments, such as systematic consultations with groups of long covid patients by the World Health Organization (World Health Organization, 2021).

17 However, there are at least some instances of successful PE described in the literature where there was initial distrust in the work of the relevant established scientists, in particular involving the citizen science approach (Ottinger, 2010). These instances are extremely valuable in that they may provide clues on how to *repair* trust which has been eroded.

18 See the infamous case of Andrew Wakefield, who had received money from lawyers intending to sue vaccine manufacturers to fabricate data suggesting a connection between vaccines and autism (Goldenberg, 2021).

19 COVID-19 vaccines (at least to date) are an example of overpromising: they were initially presented as effective in preventing infection, whereas in fact they were primarily designed to prevent serious illness. When it turned out that breakthrough infections were extremely common, this seems to have affected negatively the overall public trust in these vaccines.

20 Naturally, the role of celebrities and other persons of standing can cut both ways: such role models have also been used to stimulate vaccine uptake, more recently for the COVID-19 vaccines.

21 Personal communication with Goldenberg.

22 For example, a number of scientists and medical practitioners have been posting videos on the extremely popular TikTok platform to counter disinformation about vaccines (COVID-19 vaccines in particular).

23 A promising project is “RIVER-EU”. This is a recent European project that specifically aims at understanding the barriers and enablers that prevent or facilitate vaccination uptake in communities that are typically hesitant about vaccines (e.g., women of Turkish or Moroccan descent in the Netherlands) and developing tailored recommendations for health care professionals. https://cordis.europa.eu/project/id/964353.

24 See for instance the discussion on the limited room conceded to PE in the draft plans of *Horizon Europe*, the successor of Horizon 2020 (Mejlgaard et al., 2018).
